# Quantifying the quiet epidemic: Diagnosing dementia in late 20^th^-century Britain

**DOI:** 10.1177/0952695114536715

**Published:** 2014-12

**Authors:** Duncan Wilson

**Affiliations:** University of Manchester, UK

**Keywords:** Blessed Dementia Scale, dementia, National Health Service, old age psychiatry

## Abstract

During the late 20^th^ century numerical rating scales became central to the diagnosis of dementia and helped transform attitudes about its causes and prevalence. Concentrating largely on the development and use of the Blessed Dementia Scale, I argue that rating scales served professional ends during the 1960s and 1970s. They helped old age psychiatrists establish jurisdiction over conditions such as dementia and present their field as a vital component of the welfare state, where they argued that ‘reliable modes of diagnosis’ were vital to the allocation of resources. I show how these arguments appealed to politicians, funding bodies and patient groups, who agreed that dementia was a distinct disease and claimed research on its causes and prevention should be designated ‘top priority’. But I also show that worries about the replacement of clinical acumen with technical and depersonalized methods, which could conceivably be applied by anyone, led psychiatrists to stress that rating scales had their limits and could be used only by trained experts.

## Introduction

During the latter half of the 20^th^ century, practitioners in a range of medical disciplines employed numerical rating scales to diagnose illness and differentiate the ‘normal’ from the ‘pathological’. Gerald Grob (2011: 22) summarizes the increasing use of these methods when he remarks that ‘in the last third of the twentieth century, numbers and scales became the definition of disease’. This is certainly the case with dementia, a syndrome that predominantly affects the elderly and is characterized by progressive failure of memory and intellectual functioning. During the 1960s, British psychiatrists increasingly used standard tests and numerical rating scales to portray dementia as a stable diagnostic category and to transform ideas about its causes and magnitude. Whereas dementia was often viewed as part of ‘normal’ ageing for much of the 20^th^ century, with little agreement on its pathological basis, the use of standard tests and rating scales helped psychiatrists reposition it as a common disease that was largely caused by the accumulation of amyloid deposits known as ‘senile plaques’ in the brain.^1^

The late 20^th^ century thus constitutes a critical period of transition for our understanding of dementia, but it remains largely unexplored by historians. Histories of dementia concentrate on the United States and say little about important British developments, including the use of psychometric tests and the development of rating scales from the 1960s onwards (Ballenger, 2006). General histories of psychiatry, meanwhile, neglect the growth of old age psychiatry and the growing interest in dementia during the late 20^th^ century (Shorter, 1998), while accounts of psychiatric classification and diagnosis also overlook dementia and concentrate on the development and reception of the *Diagnostic and Statistical Manual of Mental* *Disorders* (Kirk and Kutchins, 1992). This article looks to fill these historiographical gaps by charting the increasing use of standard tests and numerical rating scales, showing how psychiatrists used them to standardize diagnosis, establish clear nosological boundaries and present dementia as a prevalent neurological disease. My analysis centres largely on the Blessed Dementia Scale (BDS), which was developed in a collaborative project between the old age psychiatrists Garry Blessed and Martin Roth and the pathologist Bernard Tomlinson during the 1960s. Working in Newcastle upon Tyne, Blessed, Tomlinson and Roth used the BDS to collect information from a relative or spouse concerning a patient’s ability to deal with 28 basic personal, domestic and social tasks. The responses for these categories were used to collate a numerical ‘dementia score’, with a higher score indicating a greater degree of cognitive impairment. After post-mortem examinations, Blessed, Tomlinson and Roth found a significant correlation between a high BDS score and the number of senile plaques in the brain, which had been linked to Alzheimer’s disease since 1907 (Beach, 1987; Ballenger, 2006). This, in turn, transformed attitudes to Alzheimer’s disease, which had previously been seen as a rare and pre-senile condition but was subsequently viewed as the major cause of dementia.

I argue that old age psychiatrists used scales such as the BDS in order to assert and consolidate their professional expertise during the late 20^th^ century. This complements histories of scientific and medical professionalization, which detail how practitioners in different fields used standard techniques to enable ‘conditions for disciplinary stabilization’ by staking jurisdictional claims over particular illnesses and patient groups (Rose, 1996: 37; see also Abbott, 1988). Roth and other old age psychiatrists believed the adoption of rating scales would increase their authority by overcoming the fact that ‘psychiatrists may differ widely from one another in the diagnostic judgement they make in identical cases’ (Roth, 1967: 428). They viewed rating scales as what Theodore Porter (1995: ix) calls ‘technologies of distance’, which generate uniform results across a range of settings by ‘leaving nothing to the personal preference and initiative of the investigator’ (ibid.: 429).

Quantification here was not only designed to standardize the actions of the observer, but also standardized ‘the subject of measurement’ (Rose, 1999: 207) by producing knowledge independently of the elderly patient, whose cognitive impairment could often be ascribed to a number of conditions, or to factors associated with ‘normal’ ageing such as poor hearing or mild forgetfulness. While I am not arguing that rating scales invented the category of the dementia patient in this period, I show how they helped demarcate dementia patients from ‘normal’ individuals and those with illnesses such as depression. This, in turn, changed ideas about the prevalence of these patients in the community and fashioned them into objects of political and social concern, which helped old age psychiatrists portray their field as vital to combating what medical journals and newspapers called a ‘quiet epidemic’ (Anon., 1978: 1).

But this broad ‘disciplinization’ thesis does not tell the whole story (Rose, 1996: 57). Diagnostic rating scales often resonate with ways of thinking beyond the professional contexts of their generation and use; and it is important that we look to broader social, political and economic factors when seeking to explain why they have become central to diagnosing dementia in recent decades (Hirshbein, 2009: 96). At a general level, the development of scales such as the BDS was informed by and contributed to concerns with the health, welfare and economic costs of an increasingly elderly population. Following the Second World War, psychiatrists often argued that ‘as the proportion of old people in the community steadily increases, so they provide an increasingly high proportion of our mentally infirm population who must be cared for’ (Lewis, 1946: 150). They claimed that accurate diagnosis was vital to determining the rates of mental illness and assessing what care, if any, particular groups required.

These concerns were especially pronounced in Britain, where elderly patients in mental hospitals took up a significant proportion of long-stay beds after the National Health Service (NHS) was established in 1948 (Freeman, 1999). Psychiatrists here claimed that dementia placed the NHS, and by extension government resources, under ‘extreme pressure’ and stressed that reliable diagnostic methods were vital to the accurate ‘deployment of manpower and money’ (Kay, Beamish and Roth, 1964: 146; Lewis, 1972: 8). Their arguments resonated with policy-makers at the government’s Department for Health and Social Security (DHSS), who were keen for elderly patients to be treated in the community for as long as possible and believed that standardized tests and scales performed a vital bureaucratic function by encouraging ‘better use of resources’.^2^ With this in mind, I believe that we cannot account for the development and popularity of rating scales in Britain without appreciating how the promise of ‘more detailed and quantifiable information’ met the political desire for rationalization and efficiency in the welfare state (Roth, 1967: 428; Sturdy and Cooter, 1998).

Yet this is by no means a linear or a progressive history. During the 1970s and 1980s biochemical research raised the possibility of treating dementia, which led some psychiatrists and funding bodies to claim that improved scales were needed to identify mild cases of dementia ‘during life’ (Lishman, 1977: 3). These concerns ensured that rating scales were seen less as bureaucratic instruments and more as a crucial first step in identifying recipients of drug treatments, prompting the development of more thorough diagnostic tests from the 1980s onwards. At the same time, Martin Roth and others maintained that rating scales never provided automatic diagnosis and stressed the importance of ‘clinical judgements’ in interpreting both the symptoms exhibited by patients and the numerical data that scales generated (Roth, 1967: 431). Their arguments reflected concerns that over-reliance on standardized and impersonal methods would replace the need for trained experts, which highlights an underlying tension in efforts to promote ‘greater rigour and discipline’ in psychiatry, as in medicine and science more generally (Roth, 1967: 427; Lawrence, 1985). As we shall see, while psychiatrists endorsed rating scales in order to structure practices and acquire social approval, they simultaneously stressed their limits in order to assert the need for clinical acumen and to retain control over them.

## Categorizing dementia: Approaches and problems, 1900–50

During the late 19^th^ and early 20^th^ centuries, psychiatrists defined dementia as a state of irreversible cognitive decline that mainly affected the elderly (Berrios, 1996; Draaisma, 2009). Keen to align psychiatry with other branches of clinical medicine, they identified two pathological causes: vascular decay, known as ‘arteriosclerosis’, and the accumulation of senile plaques in the cerebral cortex. But despite highlighting these pathological markers, psychiatrists were unable to establish whether dementia was a specific disease or part of normal ageing (Beach, 1987). Although arteriosclerosis was generally considered the major cause of dementia, for instance, it was regularly found in post-mortems of elderly patients who had displayed no clinical symptoms while alive (Berrios, 1996: 192–3).

This was also true for the senile plaques that were first linked to dementia in the 1890s, and which Alois Alzheimer famously described in the post-mortem of Auguste Deter, a 55-year-old woman who had spent 4 years in hospital suffering from cognitive impairment, delusions, hallucinations and focal symptoms. Although Emil Kraepelin classified Alzheimer’s disease as a separate disorder, most psychiatrists believed it was a rare and serious form of dementia that was characterized by early onset and greater accumulation of senile plaques (Draaisma, 2009: 217). But like arteriosclerosis before it, the association between plaques and dementia was undermined in 1933, when the German pathologist N. Gellerstedt studied the post-mortem brains of 50 patients who had shown no clinical symptoms of dementia and reported that 84% contained senile plaques.

The inability to establish a clear pathological basis for dementia led some to incorporate it into a psychodynamic approach that emphasized external factors in the development of mental illness: privileging mind over matter, and focusing on family upbringing, personal stress and environment (Micale, 2002: 310; Ballenger, 2006: 36–56). In 1937, for instance, the American psychiatrist David Rothschild argued there was no clear relationship between age, the number of senile plaques and the presence or severity of dementia. Following autopsies on 24 dementia patients, aged between 66 and 100 at the time of death, Rothschild claimed ‘the brain of the 100 year old patient showed much fewer plaques…than the brains of some of the patients in [their] sixties and seventies’ (1937: 772). He also claimed that some patients exhibited ‘minimal amount of intellectual impairment though the changes in the brain, particularly with respect to plaques, were very extensive’. Rothschild concluded that when these findings were combined with Gellerstedt’s work, ‘there were enough exceptions to make one reject the idea that there could be any simple and direct quantitative relationship between clinical and pathological changes’ (ibid.: 776). Like other advocates of psychodynamic approaches, he believed pathological factors were ‘only one element in a total picture’ (Rothschild, 1942: 418). For Rothschild, the major factor in whether a person developed dementia lay in the capacity ‘to compensate for structural damage to the brain’, which he believed could be determined by hereditary and social factors, or even by ‘difficult personal problems arising in everyday life’ (ibid.: 435).

Rothschild also questioned the traditional classification of dementia. In an article for the *American Journal of Psychiatry*, he argued it was difficult to differentiate between patients whose dementia resulted from arteriosclerosis and those for whom it derived from senile plaques. While the former often developed at a later age, with more pronounced intellectual impairment, and latter cases tended to show depressive and hypochondrial symptoms, Rothschild claimed that ‘pure forms’ occurred ‘less often than mixtures of the two processes’ (1941: 333). He also argued that it was almost impossible to differentiate between dementia and other causes of cognitive impairment. To illustrate, he detailed how psychiatrists had diagnosed dementia in 16 cases where patients showed evidence of confusion and memory loss, but outlined that autopsies in all these cases showed no evidence of senile plaques or arteriosclerosis. Cognitive impairment here arose instead from the effects of brain tumours, neurosyphilis, subdural haematoma, alcoholism, schizophrenia and depression. This led Rothschild to conclude that ‘almost any mental disorder may be mistaken for a senile or arteriosclerotic process’ and it was therefore ‘difficult or impossible to make an accurate diagnosis on clinical grounds’ (ibid.: 330). Since misdiagnosis sometimes proved fatal, as in cases of lead poisoning or cancers that were classified as dementia and left untreated, he warned that ‘one must guard against a variety of pitfalls in the diagnosis of senile and arteriosclerotic psychoses’ (ibid.: 332).

Psychiatrists in the 1880s had noted that some conditions fostered dementia-like symptoms that often disappeared following treatment, labelling this phenomenon ‘pseudodementia’ (Berrios, 1996: 190). Interest in pseudodementia waned during the early 20^th^ century, but Gellerstedt’s and Rothschild’s studies prompted a renewal of work on the potential overlap between dementia and other conditions during the 1950s and 1960s. In 1959, for example, a group of Chicago psychiatrists studied 300 patients who ‘exhibited much in their behaviour suggestive of organic disease’ and reported that symptoms ascribed to dementia often disappeared following electroconvulsive therapy (ECT). Like Rothschild, they used these findings to question the categorization of mental illness in elderly patients and asserted that since ‘no two cases are the same’, it was ‘difficult to classify these disorders in accordance with the standard nomenclature of disease in general use’ (Madden *et al*., 1959: 1570).

## Constructing a ‘natural history of mental disorder in old age’: British approaches in the 1940–60s

Throughout the 1940s and 1950s, a small but influential group of British psychiatrists echoed Rothschild’s claim that ‘psychoses of the aged now appear as the leading problems of psychiatry’ (1941: 324). Foremost among these was Aubrey Lewis, professor of psychiatry at the University of London, who argued in 1945 that increasing life expectancies and the growing number of elderly patients in mental hospitals had transformed ageing into a ‘major problem’ for psychiatry and society more generally. Lewis predicted that if intake rates continued to rise, then ‘the mental disorders of the elderly’ are ‘likely to be responsible within the next thirty years for the bulk of patients admitted to mental hospitals’ (1946: 169). But he also cautioned that it was difficult to plan ahead accurately and estimate ‘bed needs’, because diagnosis and classification of mental illness among the elderly remained ‘in a mess [and] the delimitation of the problems for study is needlessly difficult’ (ibid.).

These arguments were endorsed during the early 1950s by the Hungarian-born psychiatrist Martin Roth, who trained under Aubrey Lewis in the 1940s and shared his interest in old age psychiatry. Like Lewis, Roth argued that the growing rates of mental illness in older people constituted ‘a problem of increasing magnitude’ for the NHS, which had inherited thousands of elderly patients in long-stay mental wards when it was established in 1948 (Roth, 1955: 281). He also sought to counter the widely held view that all mental illness in the elderly was an inevitable and untreatable result of ageing, pointing to evidence that patients with depression responded well to ECT. Roth argued that psychiatrists should exercise more care when diagnosing psychiatric illness among elderly patients, in order to determine whether or not their condition was treatable.

This outlook led Roth to undertake a programme of research on the classification and diagnosis of mental illness in elderly patients after his appointment as director of research at Graylingwell Hospital, in West Sussex, during the early 1950s. As he began to construct this classificatory scheme it was clear that Roth rejected the psychodynamic approaches then prevalent in the United States. Like many British psychiatrists, he endorsed Kraepelin’s view of mental illnesses as distinct entities, or ‘natural kinds’, that could be grouped according to their specific properties and outcomes (Hilton, 2005: 425; Kay, 1999: 363).^3^ Along with William Mayer-Gross and Eliot Slater, with whom he wrote an influential textbook on *Clinical Psychiatry*, Roth argued that psychiatrists could attain comparable status to clinical doctors, and prove their importance to the NHS, only by categorizing and distinguishing between specific illnesses in order to create a ‘natural history of mental disorder’ (Roth, 1955).

Roth’s enthusiasm for this classificatory scheme was clear in a 1952 paper he co-authored with John Morrissey, which dismissed Rothschild’s claims for a ‘mixed picture’ and argued that in the majority of cases dementia could be distinguished from affective disorders such as schizophrenia or depression. After studying admission records for 150 patients over 60, and following up each case after 6 months, Roth and Morrissey claimed to find ‘a clear line of demarcation between affective disorder and senile psychosis’ (1952: 71). Patients with depression, they argued, displayed sudden onset of symptoms and responded well to ECT, while patients with dementia exhibited gradual decline and proved unresponsive to treatment. In contrast to the psychodynamic belief that ‘no two cases are the same’, Roth and Morrissey asserted that dementia and affective disorders were ‘distinct nosological entities’ (ibid.: 79).

Yet Roth and Morrissey did not believe that compiling a natural history hinged solely on clinical judgements, since these reflected ‘subjective verdicts’ and often varied between individual psychiatrists (1952: 71). They argued that the clinical assessment of symptoms should be augmented by the regular use of psychometric tests and statistics, in order to standardize diagnosis between individuals, foster common understandings of mental disorders, and increase the professional authority of psychiatrists by giving them jurisdiction over a well-defined set of conditions. This approach was clear in a 1953 paper for the *Journal of Mental Science*, where Roth and Barbara Hopkins claimed that applying a set of three psychometric tests and one memory test, which became known as the ‘Roth–Hopkins test’, had allowed them effectively to distinguish dementia patients from those with affective disorders. The psychometric tests were the Vocabulary sub-test, where patients had to define certain words; the Digit Span sub-test, where patients had to repeat numbers in forward and reverse sequence; and the Progressive Matrices test, where patients had to identify the missing element that completed a certain pattern. The information test consisted of 10 questions concerned with orientation for time, place and person, and another 10 relating to well-known events, people and dates. Roth and Hopkins outlined how dementia patients consistently scored far lower in each assessment, and concluded that the ‘differing performance of patients in a standardized test situation provides objective and independent support for the differentiation between affective disorder and senile psychosis’ (1953: 449).

But Roth and Hopkins also admitted these tests had limitations when applied to elderly patients. They outlined how they had been unable to categorize several patients because it was unclear whether their test performances were due to dementia, poor hearing, or the mild confusion that often accompanied ‘normal’ ageing (Roth and Hopkins, 1953: 442). They also noted that while psychometric and memory tests helped differentiate dementia from affective disorders, the tests said little about the degree of cognitive impairment. In a later paper, Roth acknowledged that since a rigorous ‘natural history’ should link clinical symptoms to pathological data, post-mortem work was needed to establish ‘the biochemical and physiological differences between groups’ (1955: 300). This was especially necessary, he argued, in order to counter psychodynamic claims that there was no clear pathological distinction between dementia patients and normal old people.

Roth attempted to initiate post-mortem work at Graylingwell and sent brains to the neuropathologist William McMenemy. But McMenemy died before he produced any results, and Roth left Graylingwell in 1956 to take the chair of psychological medicine at the Newcastle medical school, which was part of Durham University until a 1963 reform constituted Newcastle University as a separate institution (Roth interview, 1988). Here, Roth established a research group dedicated to old age psychiatry and began a project with David Kay and Paul Beamish on the epidemiology of mental illness among the elderly population of Newcastle upon Tyne. As part of this project, which began in 1960, Kay visited 297 elderly individuals living at home, selected from the electoral register, and used the Roth–Hopkins test to ascertain whether they were free of mental illness, had an affective disorder, or had senile dementia. He then compared these findings with the results of similar tests on hospital inpatients, and used statistical methods to test for any significant differences between the two cohort groups. The results of these tests led Kay, Beamish and Roth to claim that nearly 40% of their cohort had either an organic or an affective illness, with only 20% of these being cared for in hospital or a nursing home. They also stated that over 8% of the cohort suffered from dementia, which occurred at ‘considerably higher’ rates than previously thought (Kay, Beamish and Roth, 1964: 153).

At the outset of a paper in the *British Journal of Psychiatry*, Kay, Beamish and Roth claimed that standard tests and statistical methods had allowed them to accurately estimate the rates of mental illness among the elderly population, to assess ‘the relative frequency of the different forms’ and to determine the proportion receiving care in institutions compared with those staying at home (1964: 146). They also stressed the practical benefits of this approach by arguing that: ‘In a field where the Health and Welfare Services are under extreme pressure, data such as this are an indispensible starting point for deploying the available resources to optimal advantage’ (ibid.). In a 1966 paper written with the hospital consultant Michael Hall, Kay and Roth went further and outlined how they believed resources should be allocated. They warned that if the high proportion of elderly people affected by mental illness ‘were converted into a demand for beds it would threaten to overwhelm the hospital service; and the only practicable solution is to develop programmes of care centred on a well-coordinated community service whose aim must be to maintain the old person within his own home environment by every possible means’ (Kay, Hall and Roth, 1966: 971).

These arguments struck a chord with politicians and health administrators who had advocated non-institutional treatment for elderly patients since the 1950s, in order to free up beds and save money, and looked to adopt a similar model for psychiatric services after the 1959 Mental Health Act endorsed community care (Freeman, 1999; Thane, 2000: 452). During the 1960s the government and regional health authorities consequently viewed epidemiological surveys and standard diagnostic tests as vital to calculating bed numbers, allocating staff and planning the needs of psychiatric patients in the community (Evans, 2013: 13–14; Hilton, 2008: 308). Although politicians generally ignored the psychiatric needs of elderly patients for much of the 1960s, pressure from figures such as Martin Roth and public concerns surrounding institutional care led the DHSS to produce a paper on *Services for Mental Illness Related to Old Age* in 1972.^4^ This began by endorsing efforts to ‘replace the large mental hospitals’ with community-based facilities. It then repeated Kay, Roth and Beamish’s claim that nearly 10% of individuals over 65 had dementia, but stressed that most moderate or mild cases could generally be ‘looked after satisfactorily at home’ (Department of Health and Social Services, 1972: 1). Crucially, it also echoed a central claim of their work when it portrayed accurate diagnostic methods as ‘a fundamental requirement’ that helped ensure patients received ‘the most appropriate placement and pattern of care and treatment’ (ibid.: 2–3).

This overlap can be partly explained by the fact that some old age psychiatrists played a major role in writing the DHSS paper (Hilton, 2005: 427). But it also demonstrates how civil servants and DHSS medical officers viewed Roth’s Newcastle group as a ‘unique resource for research into dementia’ that helped in planning services and enhanced the prospects of community care.^5^ Their enthusiasm was evident during the early 1970s, when Roth and his colleague Klaus Bergmann requested money for a new project that would ‘provide information on the early stages of dementia and about the personnel most likely to make a contribution toward sustained viability of the elderly in the community’.^6^ Roth and Bergmann claimed they would use standard tests to identify cases of dementia before patients reached a ‘crisis stage’ and required hospitalization. This, they continued, would increase the possibility for community treatment and reduce ‘critical stresses on existing provisions of the Health and Social Services’.^7^ Roth and Bergmann’s proposal was favourably received by civil servants, who replied that ‘evaluation of alternative patterns of care for presented dementia cases currently ranks as our top priority’, and requested information on whether the project would ‘show if early intervention was more economic and would lead to better use of resources’.^8^

But the Principal Medical Officer at the DHSS questioned whether Roth and Bergmann’s new project was feasible, since it was often difficult to distinguish the early stages of dementia from depression and the mild forgetfulness that regularly accompanied normal ageing.^9^ These misgivings led the DHSS to warn Roth and Bergmann that it would fund a new long-term study only once researchers ‘sort out problems of definition and their interpretation’.^10^ Although Roth and Bergmann expressed disappointment at the decision, they conceded that the early stages of dementia, depression and normal ageing ‘did not appear as discrete entities’ and sometimes ‘overlapped considerably with each other’.^11^

This problem had already been noted by Roth and Hopkins (1953: 442) and David Kay later admitted to confronting it during his epidemiological work. Kay conceded that poor responses in the Roth–Hopkins tests, which he often ascribed to early dementia, might also be due to depression, normal ageing, or even a ‘poor background of education and intelligence’ (2000 interview: 247). As Kay, Beamish and Roth acknowledged, standard tests offered little clarity in these situations and diagnosis thus hinged on the ‘unavoidably arbitrary’ judgement of the consulting psychiatrist (1964: 151). What was more, when Kay followed up the cohort several years later he observed that some individuals whom he had diagnosed with early dementia exhibited no further decline, which caused the DHSS to wonder whether ‘the numbers derived from the Newcastle study represent substantial exaggerations of the true position’.^12^

## Quantifying dementia: The ‘Newcastle study’ and the Blessed Dementia Scale

As Roth and Bergmann pointed out in a letter to the DHSS, researchers at Newcastle had already undertaken a project that sought to better quantify the severity of dementia, including mild and moderate cases, in order to establish what relationship existed between pathological change and the degree of cognitive impairment. This project, which was funded by the Medical Research Council (MRC) and became known as the ‘Newcastle study’, was essentially a resumption of the aborted Graylingwell research that aimed to link psychiatric assessment to post-mortem analysis. It took shape around 1960, when Roth asked the psychiatric registrar Garry Blessed to assess the symptoms of elderly patients in the local mental hospital and then assess a control group who had been admitted to the general hospital with physical illness (Roth interview, 1988; Blessed interview, 2013).

Blessed used a modified version of the Roth–Hopkins test to check that the control group was free from psychiatric illness and to classify the mental ward patients as having either functional disorders or dementia. But while these psychometric tests helped Blessed categorize patients, they provided no quantifiable information on the level of cognitive impairment. This led him to design a rating scale that aimed to reflect how ‘demented people have a catalog of symptoms and disabilities, and at a very simple level, those who are just starting with dementia don’t have very many while those who are 2–3 years down the line have a lot’ (Blessed interview, 2000: 88). While the first application of rating scales in psychiatry dated back to soon after the First World War, by the 1960s the scales were increasingly used (Blessed interview, 2013). But psychiatrists rarely used scales to determine severity or differentiate conditions from one another, and generally used them to assess possible improvements in symptoms during clinical trials for new psychotropic drugs (Hamilton, 1976: 347).

This meant that Blessed had little to draw on when he designed his new test. Perhaps unsurprisingly, then, his 28 scoring criteria were derived from well-known symptoms of dementia: including an inability to dress unaided, to recall events, or to eat correctly with utensils, a tendency to wander or get lost in familiar surroundings, incontinence, and personality changes such as ‘increased petulance’ and ‘purposeless hyperactivity’ (Blessed, Tomlinson and Roth, 1968: 809). Blessed also decided to bypass the problems that arose when psychiatrists interviewed elderly patients by seeking information from spouses, relatives, or carers. He collected information relating to a 6-month period because he believed this was long enough to distinguish the slow decline associated with dementia from the sudden onset in affective disorders. After interviews, Blessed translated the responses into a numerical score. He gave total incompetence in each activity a score of 1, partial or variable incapacity a score of 0.5, and full capacity a score of 0. He then calculated each patient’s ‘dementia score’ by adding the numbers for each item: the minimum score was 0, indicating fully preserved capacity, and the maximum score was 28, indicating severe incapacity. If patients survived longer than 6 months, Blessed or nursing staff repeated the test and recorded any significant changes (Blessed interview, 2013).

After each of the control and psychiatric patients died, Blessed approached the family and sought permission for an autopsy. If the family agreed, the pathologist Bernard Tomlinson removed the brain and placed it in a solution of formol saline for 6 to 12 weeks. Tomlinson then sectioned the cerebral cortex and analysed tissue slices using a new computer program that allowed accurate measurement of neural cells and plaques in a given field. Without knowing the patient’s dementia score, he counted the number of plaques in several fields and calculated a mean plaque count for each brain (Tomlinson interview, 1992). When the post-mortem observations were combined with BDS scores, Blessed, Tomlinson and Roth claimed to find a ‘highly significant correlation between mean plaque counts and scores for dementia’ (1968: 804), which they argued dealt a significant blow to the psychodynamic belief that pathological change was of little significance to cognitive decline.^13^

Although Blessed, Tomlinson and Roth observed senile plaques in the brains of controls and patients with affective disorders, who had mean plaque counts ranging from 1 to 5, patients diagnosed with dementia had a far higher mean plaque count of 20.85, and there was a general ‘tendency for performance on the test to decline with increasing plaque formation’ (1968: 803). This led them to portray dementia as an ‘accelerated and intensified’ version of seemingly common ‘pathological changes associated with senescence’ (Roth, 1971: 2, 6). Publishing their results in a 1968 paper for the *British Journal of Psychiatry*, Blessed, Tomlinson and Roth argued that while there was no specific pathological change that differentiated dementia patients from the controls and patients with depression, ‘the difference between “senile dements” and those other subjects reflect a quantitative gradation of a pathological process common in old age, rather than qualitative differences’ (1968: 805).

So while ‘normal’ ageing was inherently pathological in this view, an individual developed dementia only once ‘the degenerative process measured by plaque counts develops beyond a certain threshold point’ (Blessed, Tomlinson and Roth, 1968: 805). In a later paper, Tomlinson, Blessed and Roth again highlighted the significance of senile plaques by rejecting the long-standing view that arteriosclerosis was the major cause of dementia. After reassessing all the brains from patients diagnosed with dementia, including those with large areas of vascular decay that were excluded from the initial study, they claimed that senile plaques accounted for dementia in over 50% of cases, with only 17% being due to arteriosclerosis alone and 18% showing a mixed picture (Tomlinson, Blessed and Roth, 1970).

This work did not attract instant attention. There was little interest when Blessed and Tomlinson presented a preliminary report at the 1965 meeting of the World Psychiatric Association; and when Roth chaired a 1970 CIBA Foundation symposium on ‘Alzheimer’s Disease and Related Conditions’, many participants were reluctant to accept that senile plaques were the major cause of dementia (Blessed interview, 2013; Wolstenholme and O’Connor, 1970). In 1975, however, members of the MRC Psychiatry Committee, including Martin Roth, appointed a subcommittee to make recommendations for future research on dementia. The subcommittee’s report, published in 1977, was clearly informed by the work undertaken in Newcastle when it presented dementia as a specific ‘disease state’ (Lishman, 1977: 1). At the outset of the report, the MRC subcommittee cited Kay, Beamish and Roth’s work when it claimed that dementia ‘constitutes one of the biggest problems facing the health and social services today, largely as a result of the increased number of people achieving longevity’. Increasing life expectancies, the report argued, ensured ‘the urgency of the research challenge is evident, and has a bearing on the present crises and shortages in healthcare’ (ibid.).

These claims underpinned the report’s first and major proposal: that the government and funding bodies should designate research into dementia ‘as areas of high priority’ (Lishman, 1977: 21). The subcommittee considered Blessed, Tomlinson and Roth’s work as one of the ‘promising leads’ that shed light on the major causes of dementia and helped differentiate it from normal ageing. But it nevertheless claimed more research was vital and called for greater investment in old age psychiatry and ‘joint clinical-laboratory enterprises’ along the lines of the Newcastle study (ibid.). This was vital, the subcommittee argued, in order to determine which biochemical, genetic, or environmental factors caused the accumulation of senile plaques and ‘sets the pathological process in motion’ (ibid.: 9).

The largely positive response to this report indicates that few people, if any, now questioned the view of dementia as a distinct and widespread disease. Perhaps unsurprisingly, given how the subcommittee presented research into dementia as the solution to a healthcare ‘shortage’, the DHSS welcomed its proposals and ‘agreed that it should form the basis of a statement of joint MRC/Health Department policy’.^14^ In a similarly positive editorial, the *British Medical Journal* portrayed dementia as a ‘quiet epidemic’ and called for urgent measures to address the ‘medical neglect of this devastating and fatal illness’ (Anon., 1978: 1–2). *Nature* also claimed that more research was vital as ‘nearly 5–10% of elderly people in the UK suffer from senile dementia, about which virtually nothing is known’ (Anon., 1977: 645). A long article in the *Observer*, meanwhile, reiterated the portrayal of dementia as a ‘sad, quiet epidemic’ and informed readers that it arose mainly from cerebral degeneration rather than ‘impaired blood circulation’, as had previously been thought (Doyle, 1979: 65). This latter claim was endorsed in a letter to the *Observer* from the chairman of the Alzheimer’s Disease Society, which was established in 1979, who praised how psychiatrists were now drawing increased attention ‘to the problems of dementia’ and outlining how senile plaques were its ‘commonest underlying cause’ (Wilcock, 1979: 26).

## Critiquing the BDS: Diagnostic uncertainty, new scales and the limits of objectivity

The BDS was central to growing this portrayal of dementia as a ‘quiet epidemic’ that was caused largely by senile plaques; and Blessed, Tomlinson and Roth promoted it to colleagues as a reliable test that allowed ‘clinical evaluation to be made in a consistent manner and expressed in as precise a form as the situation permits’ (1968: 799). As before, Roth claimed standardized tests such as the BDS were vital to consolidating the expertise of old age psychiatrists. He claimed they would ‘fashion more precise and disciplined workers out of indifferent ones, will increase standards all round and will raise the general level of scientific communication within the field of psychiatry’ (1967: 437).

Like practitioners in other fields, Roth viewed classificatory schemata and standard tests as a means of ‘organizing working practices’ and ensuring psychiatrists acted in unison (Bowker and Star, 2000: 31). By the mid-1970s, there were signs that this ambition was being realized when other psychiatrists began to use the BDS. After he returned from a spell in the United States, Klaus Bergmann told Blessed that some American psychiatrists now employed the BDS in their work (Blessed interview, 2013). In Britain, meanwhile, the authors of a 1973 MRC grant proposal on ‘clinical and nosological enquiries into dementia’ claimed the BDS had ‘proved its value’ in the Newcastle study and would be one of several tests they used to assess and classify various sub-types of dementia among elderly patients in London.^15^

But uptake of the BDS was not as uniform as Blessed, Tomlinson and Roth may have wished. Researchers on a ‘US–UK Diagnostic Project’ showed that psychiatrists in different institutions employed a variety of standard tests, with different scoring criteria, when they assessed patients. This was certainly the case with dementia, where psychiatrists could choose between the BDS or a newer Mini Mental State Examination, which had been developed by the American psychiatrist Marshal Folstein in the mid-1970s (Folstein interview, 2000). What was more, the Diagnostic Project researchers claimed the choice of test might influence diagnosis: with diagnostic statistics varying across different locations, and recorded cases of arteriosclerotic dementia higher in the United States than in Britain (Lewis, 1972: 137–40). Although the researchers conceded that social and environmental factors might influence these differences, they noted that diagnostic rates became more uniform when different psychiatrists all used the same test. These findings led David Kay to warn the MRC that the classificatory scheme proposed in the ‘clinical and nosological’ project might have limited impact, as psychiatrists used different tests for dementia and ‘diagnoses diverge considerably’.^16^

By the late 1970s, scales such as the BDS also came under fire for their inability to distinguish early and mild cases of dementia. The MRC subcommittee claimed they ‘tend to lack sensitivity’ and recommended that psychiatrists should develop newer scales to enable ‘clearer demarcation of disorders from their surrounding territories – from other disease processes which mimic their clinical picture and from the “natural” processes of senescence’ (Lishman, 1977: 14, 3). It stressed that developing new scales had become a priority thanks to biochemical research that raised the possibility of treating dementia and, in doing so, increased the importance of ‘making an accurate diagnosis during life’ (ibid.: 3).

The possibility of drug treatments for dementia increasingly led psychiatrists to view rating scales not as a bureaucratic tool that helped allocate resources, but as a crucial precursor to ‘intervening in the disease process’ (Lishman, 1977: 1). One of the earliest findings to raise this possibility emerged from another MRC project in Newcastle, involving the biochemist Elaine Perry, the neuropathologists Robert Perry and Peter Gibson, and Garry Blessed, Bernard Tomlinson and Klaus Bergmann. This group observed that brain tissue taken from patients who had been diagnosed with dementia contained lower than normal levels of the neurotransmitter acetylcholine, which had been shown to play a role in memory and learning. In a 1978 paper for the *British Medical Journal*, they claimed that levels of acetylcholine ‘decreased significantly as the mean plaque count rose’ and that the reduction in acetylcholine ‘correlated with the extent of intellectual impairment’ measured by standard tests (Perry *et al.*, 1978: 1457). Although Perry and colleagues cautioned that more research was needed to ascertain the precise relationship between acetylcholine and dementia, they nevertheless predicted that efforts to correct or prevent its depletion ‘may provide a basis for therapeutic regimens’ (Perry *et al.*, 1977: 189).

Calls for improved rating scales increased as this ‘cholinergic hypothesis’ took hold in the 1980s (Moreira, 2009). In 1982, a team of American psychiatrists designed a new and extensive Clinical Dementia Rating (CDR) that used elements of existing scales, including the BDS, to collect information from a ‘collateral source’ and then subjected patients to a battery of tests in order to assess their memory, orientation, problem-solving, domestic routine and personal habits (Hughes *et al.*, 1982: 566). These psychiatrists argued that although the CDR took longer to administer than older tests, it enabled clinicians to determine more accurately whether patients had mild, moderate, or severe dementia. They also claimed it provided less scope for disagreement, with ‘independent reviewers’ who watched videos of the tests corroborating the initial diagnosis in 117 out of 123 cases (ibid.: 567). More comprehensive tests such as the CDR were vital, they concluded, since ‘potential treatments require that this assessment be made during life’ and it was no longer sufficient to reach a definitive diagnosis, as Blessed, Tomlinson and Roth had done, by validating test scores against post-mortem findings (ibid.: 566).

Similar claims were notably made by Martin Roth, who had left Newcastle to take the chair of psychological medicine at the University of Cambridge in 1977. Roth now argued that prompt diagnosis of dementia had become ‘an urgent necessity’ following ‘recent developments which have opened the possibility of biochemical treatment’ (1980: 212). Shortly after moving to Cambridge, he drew up plans for a new test to facilitate accurate diagnosis while patients were still alive. As part of a large grant proposal on ‘multidisciplinary enquiries into senile dementia’, submitted to the MRC in 1978, Roth claimed that he and colleagues would develop a new assessment schedule that overhauled the ‘relatively crude’ BDS and ‘should make it possible to depict demented patients and to discriminate them from other psychiatric patients and “normal” groups of patients with more sharpness and precision than has been possible in the past’.^17^

The award of this grant led to the development of the ‘Cambridge Mental Disorders of the Elderly Examination’, or CAMDEX. In a 1986 paper, Roth and colleagues outlined how existing scales had performed a vital bureaucratic function: providing ‘a basis for social and healthcare planning’ by ‘separating groups of patients into “demented” and “non-demented” or “functional” groups’ (Roth *et al.*, 1986: 698). But they also stated these tests did not adequately grade the severity of dementia or differentiate mild cases from normal ageing and ‘clouded and delirious states’ (ibid.: 699). The CAMDEX, Roth, Tym *et al.* argued, remedied ‘gaps in the existing standardised interviews and scales’ by combining informant interviews, detailed psychometric tests, physical examinations and new CT scanning methods, which allowed clinicians to observe brain pathology and attempt a definitive diagnosis while the patient was still alive (ibid.: 700).

The development of CAMDEX and the CDR illustrates how dissatisfaction with existing methods and the prospect of drug treatments led to new diagnostic procedures for dementia by the mid-1980s. These more thorough assessments are still commonly used, and old age psychiatrists estimate that dementia can be accurately identified in 90% of cases when rating scales, psychometric tests and imaging methods are combined (Burns, Byrne and Maurer, 2002: 166). Yet the remaining 10% demonstrates that psychiatrists still encounter ‘the difficult issue of whether these criteria can distinguish between patients with no dementia and those with minimal or questionable dementia’ (Ballard and Bannister, 2005: 25; Lock, 2013: 54). This is made clear in a recent psychiatric textbook, which warns that ‘as there are no satisfactory methods for distinguishing patients with progressive decline from those who continue to exhibit a static degree of mild impairment or improve, it is difficult to see how any clinical diagnostic criteria could distinguish these patients from those in the early stages of a dementia process’ (Ballard and Bannister, 2005: 26). Despite the development of new rating scales and visual methods, then, the boundaries between what Martin Roth called ‘senile dementia and its borderlands’ remain blurred, and psychiatrists acknowledge that ‘clinical judgements’ remain vital in these instances (ibid.: 27; Roth, 1980).

But psychiatrists had long maintained that clinical judgements do not simply underpin diagnosis in borderline cases. In 1942, for example, M. B. Brody warned that psychometric tests and rating scales could never provide automatic diagnosis, since ‘the examiner must decide in each and every case the validity of using [them] and the meaning of the result’. Brody concluded that the value of the results obtained from tests and scales, ‘like that of all clinical instruments, depends to a great extent on the ability of the user’ (1942: 326). Martin Roth made similar claims as psychiatrists increasingly began to use rating scales. During the late 1960s, he reminded colleagues that individual judgements remained vital both to selecting the appropriate scale and to interpreting data once it had been used. ‘Specialised scales still require a preliminary psychiatric diagnosis to provide some guarantee that the scale in question has relevance for the patients to be investigated’, Roth argued, while the scores they generated only made sense when ‘validated against clinical judgements’ (1967: 430). Although he regularly endorsed standard tests and rating scales, Roth stressed they were no substitute for ‘the clinical form of inquiry’ and should merely be seen as ‘safeguards to ensure that standards of precision, comprehensiveness and logical inference are maintained at a high level’ (ibid.: 435).

Like others in the sciences and medicine, Roth believed that trained judgement was needed to interpret the results produced by seemingly objective and impersonal tools such as X-rays, thermometers and diagnostic rating scales. These arguments reflected fears that dependence on standard instruments and tests would undermine the need for professional experts, with anyone seemingly able to generate results provided he or she followed the necessary protocols (Blessed interview, 2013; Lawrence, 1985; Daston and Galison, 2007: 309–62). This was clear when Roth argued that if there were no longer a role for clinical acumen, then ‘psychiatrists should depart to make way for psychologists and social scientists armed with appropriate rating scales and measures’ (1967: 436). Roth asserted the importance of trained judgement by comparing psychometric tests and rating scales with compasses and rulers. Although they helped psychiatrists draw their boundary lines with greater precision, he argued, ‘much depends on the steadiness and practice of the hand’ (ibid.: 437). These claims highlight an inherent tension in the development and promotion of rating scales, which helps explain why Roth and others continued to present diagnosis as a complex and often ambiguous process. While old age psychiatrists promoted rating scales for professional reasons, to structure practices and acquire social approval, the same motives also led them to dwell on their limitations and warn that ‘objectivity is never an absolute’ (Wittenborn, 1967: 387).

## Discussion

Historical accounts often attribute the ‘rediscovery’ of Alzheimer’s disease, and its portrayal as the leading cause of a dementia epidemic from the 1960s onwards, to ‘government and medical recognition of aging populations and their impending burden on society’ (Lock, 2013: 22; Katzman and Bick, 2000). While it is hard to dispute this thesis, there is certainly more to say about how this ‘rediscovery’ illustrates complex dynamics between psychiatry, politics and society (Pickersgill, 2012). In his work on ‘mad travellers’ and transient mental illness, Ian Hacking presents a useful model for appreciating how the mutual interplay between several factors leads doctors, politicians, patients, the media, etc., to draw attention to specific diseases in certain times and places. He warns against privileging one single factor when documenting the emergence of psychiatric conditions and instead uses the notion of an ‘ecological niche’ to capture ‘the manifold elements that make a new diagnosis possible’ (1998: 86). For Hacking, an ecological niche encompasses 4 historically specific and interacting elements that allow diagnostic categories to emerge or thrive: an existing medical framework or ‘taxonomy of illness’; a set of broad concerns that ensure certain symptoms become problematic; a way of recording these symptoms and demarcating them from ‘normal’ behaviour; and a means of resolving social, political, or professional problems by emphasizing particular disease states (ibid.: 80–2).

This article has shown how late 20^th^ century Britain provided a particularly good ecological niche for the rediscovery of Alzheimer’s disease and the presentation of dementia as a disorder that was distinct from ‘normal’ ageing.^18^ Awareness of the burden that ageing populations placed on society fulfils the second of Hacking’s elements, in that it was a broad concern that ensured the symptoms of dementia became increasingly problematic. But it was only one element in a complex picture that also included efforts to fashion old age psychiatry into a respected medical field, largely by fitting dementia into an existing ‘natural history of mental disorder’, as well as a political desire to allocate resources in the welfare state and, when possible, to treat elderly patients in the community rather than long-stay hospitals. This evokes the fourth element of Hacking’s ecological niche, with old age psychiatrists and politicians believing they could resolve problems by categorizing dementia as a distinct disease: whether increasing professional status, planning medical and social services, or saving money. We must not lose sight of these additional, yet often overlooked, motivations when explaining the ‘rediscovery’ of Alzheimer’s disease in the 1960s and 1970s.

The interaction between political and professional factors here, as elsewhere, was mediated by a ‘heterogenous assemblage of material and symbolic elements’ (Pickersgill, 2012: 329) that included clinical textbooks, funding applications, debates around ‘practical planning’ of health services (Roth *et al.*, 1986: 698), and diagnostic rating scales. Scales such as the BDS were crucial to meeting, and also linking, these professional and political aspirations. They provided an apparently standardized means of recording symptoms and helped psychiatrists portray dementia as a prevalent disease, while they simultaneously appealed to politicians and health administrators for whom estimating rates of dementia had become ‘top priority’. The popularity of the quantifiable and biological model the BDS underpinned was evidenced by the fact that competing psychodynamic approaches ‘never gained a foothold in Britain’ (Freeman, 1999: 6), while the ‘anti-psychiatry’ movement that emerged in the 1970s did not dispute the biological basis of dementia in the same way as it did for schizophrenia and other conditions. Although the BDS was increasingly criticized by the 1980s, thanks to the promise of drug treatments and demand for more ‘sensitive’ scales, it had nevertheless served its purpose by underpinning a powerful cognitive frame around which several groups, including funding bodies and new patient associations, identified common interests and formulated policies (Fox, 2000: 211).

We can therefore view diagnostic rating scales, including those no longer in common use, as vital components of what Alan Blum calls the ‘imaginary of dementia’: which is the medicalized view of neurological decline that, he argues, works to compensate for the often ‘unfathomable experience’ of dementia patients by ‘asserting “the brain did it!”’ (2012: 109–10; see also Lock, 2013: 14–15). Histories of scales such as the BDS can deepen our understanding of why this world-view first emerged and became so influential, showing how its existence depends on various material practices and tools. We have seen that portrayals of dementia as a ‘quiet epidemic’ were promoted and gained traction because they served and linked the interests of several groups, including old age psychiatrists, politicians, healthcare administrators and patient associations (to say nothing of the pharmaceutical firms which have invested heavily in the still unrealized search for drug treatments since the 1980s). This ‘medical imaginary’ became paradigmatic, in other words, not because dementia was inherently quantifiable but because, to quote Theodore Porter, certain groups made it quantifiable ‘the better to manage it’ (1995: 213).

## Notes

The research on which this article is based was funded by a Wellcome Trust Programme Grant in the History of Medicine: number 092782. I am grateful to the Wellcome Trust for its continued support.

1. I have put the term ‘normal’ ageing in quotes because geriatricians and old age psychiatrists have rarely, if ever, held consensus on what normal ageing is and how it can be measured. This was clear when one old age psychiatrist referred to ‘normal ageing, whatever that is exactly’ during an interview. See Kay (2000 interview: 248). On how perceptions of ‘normal’ ageing are influenced by culture and politics, see Lock (2013: 42–4, 223).

2. V. J. M. Poole to K. Bergmann (January 1976), National Archives MH166/1305.

3. The term ‘natural kinds’ refers to the distinct objects and processes studied by natural sciences, which feature in explanations and support inductive inferences; e.g. biological species and chemical elements. I use it here as Roth and other British psychiatrists believed there were clear and theoretically important differences between mental illnesses. For more on mental illnesses as ‘natural kinds’, see Cooper (2007: 44–6).

4. Public concerns surrounding elderly patients were largely prompted by the 1967 publication of Barbara Robb’s book *Sans Everything*, which documented institutional abuses, and were sustained by a series of inquiries into treatment at care homes during the 1970s. For more detail, see Hilton (2008).

5. Anon., Newcastle Old Age Research Unit – Meeting (November 1975), National Archives MH166/1305.

6. K. Bergmann to G. K. Matthew (March 1976), National Archives, MH166/1305.

7. K. Bergmann, Age Research Group – Psychogeriatric Project (1973), National Archives MH166/1304.

8. V. J. M. Poole to K. Bergmann (January 1976); Anon., Newcastle Old Age Research Group (1976), National Archives MH166/1305.

9. G. K. Matthew, Proposals for a Psychogeriatric Project (March 1976), National Archives MH166/1304.

10. B. A. Harrison, Proposed New Study of the Elderly Mentally Disordered in Newcastle (February 1973), National Archives MH166/1304.

11. Anon., Newcastle Old Age Group Meeting (April 1976), National Archives MH166/1305.

12. B. A. Harrison, Proposed New Study of the Elderly Mentally Disordered in Newcastle (February 1973), National Archives MH166/1304.

13. As Jesse Ballenger notes (2006: 84–5), however, the high correlation between BDS score and plaque counts owed a great deal to the control patients, who had low BDS scores and low mean plaque counts. The statistical significance of the correlation between BDS scores and plaque counts declined markedly if controls were discounted.

14. MRC Psychiatry Committee Policy Review (1976/77), National Archives FD10/332.

15. C. D. Marsden and W. A. Lishman, ‘Clinical and Nosological Enquiries into Dementias of Old Age: Appendix’ (1973), National Archives FD10/332.

16. D. W. Kay, ‘Referee’s Comments on MRC Project Grant Application’ (1973), National Archives FD10/332.

17. M. Roth, ‘Multidisciplinary Enquiries into Arteriosclerotic and Other Forms of Dementia’ , MRC Grant Proposal (January 1978), National Archives FD10/447.

18. I have not fully adopted a comparative approach in this article, but it is worth noting that several elements in this ‘ecological niche’ were specific to Britain and are not easily generalized. See Moreira *et al.* (2008) for discussion of how national particularities, including professional cultures and the organization of healthcare systems, can shape the implementation of research priorities, diagnostic categories and treatment programmes in different countries.
